# Self‐Reported Altruism Is Positively Correlated With Anxiety, Depression, and Empathy Among Gazan Healthcare Workers During War: Validation of the Arabic 9‐SRA in a High‐Stress Context

**DOI:** 10.1002/hsr2.71599

**Published:** 2025-11-27

**Authors:** Feten Fekih‐Romdhane, Kamel Jebreen, Inad Nawajah, Tasnim Swaitti, Eqbal Radwan, Wafa Kammoun‐Rebai, Bushra M. Hammad, Mohammed Jebreen, Etimad Alattar, Walaa Safi, Sahar Obeid, Souheil Hallit

**Affiliations:** ^1^ The Tunisian Center of Early Intervention in Psychosis, Department of Psychiatry “Ibn Omrane” Razi Hospital Manouba Tunisia; ^2^ Tunis El Manar University, Faculty of Medicine of Tunis Tunis Tunisia; ^3^ Department of Computational Science Palestine Technical University – Kadoorie Hebron Palestine; ^4^ Department of Mathematics Hebron University Hebron Palestine; ^5^ Smart University College for Modern Education Hebron Palestine; ^6^ Department of Biology, Faculty of Science Islamic University of Gaza Gaza City Palestine; ^7^ Medical Research Direction Ministry of Health Tunis Tunisia; ^8^ Department of Educational Administration Palestine Ahliya University Bethlehem Palestine; ^9^ Department of Business Administration Faculty of Commerce Mansoura University Mansoura Egypt; ^10^ Ministry of Transport and Communications Ramallah Palestine; ^11^ Social and Education Sciences Department, School of Arts and Sciences Lebanese American University Jbeil Lebanon; ^12^ School of Medicine and Medical Sciences Holy Spirit University of Kaslik Jounieh Lebanon; ^13^ Applied Science Research Center Applied Science Private University Amman Jordan

**Keywords:** altruism, healthcare workers, psychometric properties, self‐report altruism scale, war

## Abstract

**Background and Aims:**

This study had the main purpose of investigating the psychometric properties of the 9‐item Self‐Report Altruism scale (9‐SRA) in its translated Arabic‐language version among Palestinian HCWs in Gaza during war times.

**Methods:**

This cross‐sectional study was performed between January 15 and April 15, 2025, among 615 HCWs at several hospitals and primary health care centers in the Gaza Strip.

**Results:**

Our data adequately fitted this original hypothetical one‐factor model, with excellent internal consistency reliability coefficients (Cronbach's *α* = 0.92). The assumption of measurement invariance across sex groups of the Arabic version of the 9‐SRA was supported. Empathy scores were significantly and positively correlated with altruism scores. Finally, higher depression and anxiety were significantly associated with lower altruism.

**Conclusion:**

Our research offers robust evidence in terms of reliability and validity to support the use of the 9‐SRA instrument to evaluate self‐reported altruistic behavior among Arabic‐speaking HCWs, especially in the context of war.

## 1 Introduction

According to the United Nations Human Rights Office, an estimated 136 strikes on and near 27 hospitals and 12 other medical facilities occurred by December 2024 [1]. This has led to unprecedented challenges in providing care to those in need, leaving the already vulnerable civilian population in Gaza with very limited access to healthcare services and contributing to an exacerbation of their distress. However, despite all the chaos, hardship and death brought forth during wartime, HCWs have been on the front line, working tirelessly and risking their own lives to save others. In such circumstances, the question needs to be raised as to what might encourage HCWs to continue working and keep committed to saving lives in war settings. One potential answer may be altruism.

There is no consensual definition of altruism, but it is generally recognized as a voluntary and intentional behavior that benefit others [2], or self‐sacrificial behaviors enacted for the benefit of others that is performed without any expectation of compensations or external gains [3]. According to socio‐psychological theories, motivations for prosocial behavior can be either altruistic or egoistic [4, 5]. Altruistic behavior refers to prosocial behavior that is driven by a genuine desire to benefit others with no expectations of benefiting the self [6]. Over the past years, this dichotomized view has been questioned and debated by researchers who claimed that purely altruistic behavior may not exist and is rather stemming from egoistic motivations inherent in desiring to feel good about oneself, or to avoid negative feelings such as guilt, anxiety, or sadness [7]. Altruism is regarded as a powerful force that is unique to humans [8]. Research suggests that altruistic behaviors is subject to large individual variations [9], and might have heritable components [3]. Historically, altruism has always been described as part of the healthcare profession and one of the values that mirrors the standard of healthcare, involving a prioritization of the needs of others and a willingness to make sacrifices for them [10]. Altruism is suggested to be driven by the person's internal values rather than financial‐ or social‐reward expectations; and highly altruistic individuals are more inclined to choose nursing as a career [11]. It is reported that altruism allows and motivates HCWs to sacrifice themselves for the patient and be able to tolerate the most challenging situations [10].

The increasing number of disasters has sparked a line of research focused on understanding whether healthcare workers would continue to fulfill their work duties even when their personal safety is threatened by a disaster [12]. There is some evidence to suggest that, contrary to what was expected, nurses' willingness and dedication tended to increase during crises circumstances, such as the initial phase of the COVID‐19 pandemic [13, 14]. Furthermore, although a large body of literature agrees that engaging in altruistic behaviors confers instant and long‐term benefits for physical and mental health [15, 16, 17, 18], another stream of research has suggested that altruism can also confer more negative mental health outcomes (anxiety and depressive symptoms) when altruistic activities cannot be achieved under particular circumstances [19]. However, the majority of the research available on HCWs' experiences of working during the war mostly focused on the dire consequences in terms of mental health so far, whereas few studies have explored the topic from an altruistic perspective. HCWs' obligations or motivations to act altruistically in extreme conditions such as war need to be known and understood to help preserve health system capacity and resources in most difficult times. To achieve this, appropriate and well‐validated measures that are suitable to this target population need to be available.

### 1.1 Measurement Instruments of Altruism

Altruism has been a focal construct in the research on human nature and behavior in several scientific disciplines, including economics, management, marketing, decision sciences, biology, environmental sciences, sociology, psychology and public policy [3]. Consistently, multiple attempts have been made to operationalize and measure altruism to explain human behavior, and specifically prosocial behavior [2, 20, 21], as well as understand its origins and effects. Because of its abstract nature, its assessment as a high‐order construct has been challenging, and two principal approaches were adopted. The first one is a behavioral experimental‐based approach using experiments and games to detect altruism (such as the Dictator game [22] or the Faith game [23]. The second one is a survey‐based self‐report approach, with the most commonly accepted and used measure being the Self‐Report Altruism (SRA) scale [9]. The scale is simple and easy‐to‐administer, and was shown to accurately distinguish altruists from non‐altruists based on 20 statements reflecting respondents past behavior (e.g., “I have given money to a charity”). In the parent validation study, the SRA demonstrated solid psychometric properties, including high internal consistency and showed evidence for validity through positive correlations with other variables including organ donation, social responsibility, prosocial individual values, and empathy [9]. In this regard, the empathy–altruism hypothesis by Batson [24, 25] posits that empathic concerns (which are other‐oriented emotional reactions congruent with and produced by the perceived welfare of a person in need), generate altruistic motivations aiming at decreased that need. Later, the scale was translated into and validated across different languages and contexts, including Colombia [26], China [27, 28, 29], Japan [30], Korea [31], India [32], Iran [33], and Spain [34]. In all validation studies, good psychometric properties of the different linguistic versions of the SRA scale have been consistently reported in various populations and parts of the world, thus allowing to compare the results obtained with samples from different countries. However, due to its relative length, its use can be quite inconvenient, particularly in combination with other constructs, which makes the survey time‐consuming and respondents reluctant to participate. This issue can be particularly burdensome for research involving HCWs based within war settings.

More recently, Manzur & Olavarrieta [35] developed and validated a simplified version of the SRA scale composed of only 9 items in a research following a three‐phase design (scale simplification, pre‐test, then final validation). Their findings indicated that a two‐factor solution of the 9‐SRA showed the most acceptable fit levels, and was therefore retained as the model that best represents the structure of the altruism construct, with satisfactory internal consistency reliability (Cronbach's *α* = 0.77). The first factor is called “Charity” and it has four items (e.g., “I have done volunteer work for a charity”), while the second factor is called “helping people” and it has five items (e.g., “I have offered to help a person with a disability or elderly stranger across a street”). The Convergent validity was demonstrated by showing that all nine items converged and loaded into a higher‐order factor. Besides, 9‐SRA scores were significantly and positively correlated with donation behavior, supporting predictive validity of the scale [35]. This shorter version of the SRA scale allows reducing completion time could be more suitable in some circumstances, such as research performed in war zones. However, to date, there is a general lack of evidence to support its psychometric properties in HCWs in the context of war.

### 1.2 Rationale and Objectives

There is a lack of psychometric information on altruism measures in HCWs in general, and in those who remain committed to alleviating the suffering of people in need of care during challenging situations in particular. An examination of the psychometric properties of the 9‐SRA for the first time in HCWs working under attack in a war zone may help us to gain insight into the suitability of the scale in a unique, high‐stress applied context. In addition, as this is among the few studies to measure altruism among HCWs' in an Arab‐Palestinian context, the findings can offer new insights into how societal values in a non‐Western collectivist country and culture shape altruistic tendencies. Indeed, the SRA was developed, validated and widely used in Western, politically stable and individualist societies, which are characterized by prioritizing or emphasizing the individual over the entire group [9]. Therefore, its applicability in other cultures and nations still needs to be confirmed. In this context, we performed this study with the main purposes of: (1) investigating the psychometric properties of the 9‐SRA in its translated Arabic‐language version among frontline healthcare workers amid war in Gaza, and (2) examining associations between altruism, depression, anxiety, and empathy in this high‐stress, real‐world setting. The study hypotheses were the following: (1) confirmatory factor analysis will support the unidimensional factor structure of the scale; (2) the factor structure will be invariant across sex; (3) the scale's Cronbach's alpha will be of more than 0.7, indicating good internal consistency; (4) the scale will correlate significantly with higher depression/anxiety and dispositional empathy.

## 2 Methods

### 2.1 Design

We conducted a cross‐sectional study during a 3‐month period, from January 15th to April 15th 2025, at several hospitals and primary health care centers in the Gaza Strip including Al‐Ahli Arab Hospital, European Hospital, Shuhada Al‐Aqsa Hospital, Al‐Shifa Hospital, Kamal Adwan Hospital, Al‐Dorra Hospital and UNRWA healthcare centers. All healthcare facilities where participants' recruitment took place have been under intermittent missile attacks since the war started in October 2023, including at the time of the survey. The ethical approval for this project was granted by the Ethical Research Committee at Smart University College for Modern Education, Hebron, Palestine (Reference 12‐2024). All methods were performed in accordance with the relevant guidelines and regulations (in accordance with the Declaration of Helsinki).

### 2.2 Sample

The target population consisted of healthcare personnel (nurses, physicians, health professionals, medical transportation personnel, and others). Eligibility criteria were the following: (1) being aged 18 years and over, (2) being part of the health facility staff, and directly involved in providing care to patients, (3) being able to read and understand Arabic, and (4) willing to participate. Those who did not meet the inclusion criteria or declined to participate, and HCWs who were outsourced at time of recruitment were excluded. The data were collected using a convenience sampling approach online method to confer greater chances of reaching out to potential participants. The investigators approached all participants and asked them to voluntarily complete an anonymous online survey, which was created using Google Forms software. The link to the survey was sent to HCWs through social media groups comprising only medical HCWs from Gaza, and also distributed via email. It included the study objectives and consent information on the first page. Implied consent was considered to have been given when respondents clicked “Yes” to the question “Do you consent to take part in this study?”, and were subsequently invited to complete the survey questions.

### 2.3 Minimal Sample Size Calculation

For the confirmatory factor analysis of the 9‐SRA to be conducted, previous evidence recommended that a minimum sample ranging from 3 to 20 times the number of items is required [36]. Therefore, a sample between 27 and 180 participants was considered as appropriate.

## 3 Measures

The online questionnaire gathered data about participants' age, sex (male vs. female), marital status (single, divorced, widowed, married), occupation (physician, nurse, other healthcare worker), workplace (hospital vs. primary health care center). In addition, the three below mentioned measurements were administered to all participants.

### 3.1 The 9‐item Self‐Report Altruism (9‐SRA) Scale

This scale is composed of nine items (e.g., “I have made change for someone I did not know”). Each item has five response options (never, once, more than once, often, and very often) that are scored on a scale from 1 to 5. Total altruism scores are calculated by summing scores on all nine items, with greater scores reflecting greater altruistic behaviors. A cut‐off score of 28/45 or above was considered as indicative of high altruism [35]. To produce an Arabic version of the 9‐SRA, we followed a process that adhered to international guidelines [37]. The forward and backward translation method was applied to the 9‐SRA. First, the original English version of the scale was translated into Arabic by a native Arabic‐speaking translator from Tunisia. Afterward, a Lebanese psychologist with full working proficiency in English, who was blind to the original scale, translated the Arabic version back into English. Then, the source and back‐translated English versions were compared and critically reviewed by a panel of experts to identify and resolve any discrepancies, thus ensuring semantic and conceptual equivalence of items as well as the usability and applicability of the whole scale. Finally, a pilot study of the Arabic version was conducted with a sample of 30 adults to verify cultural suitability and comprehensibility. No changes were deemed necessary after pilot testing.

### 3.2 Patient Health Questionnaire (PHQ‐4)

This brief scale is composed of four items that are used to evaluate symptoms of depression (e.g., “Feeling down, depressed, or hopeless”) and anxiety (e.g., “Feeling nervous, anxious or on edge”) throughout the previous 2 weeks. It consists of two sub‐scales: Anxiety and Depression. It consists of a 4‐point Likert scale, with 0 denoting “not at all” and 3 denoting “almost every day.” The addition of the scores for each of the four PHQ‐4 items determines the final score [38]. The Arabic validated version was used [39], with a Cronbach's alpha of (*ω* = 0.87/α = 0.86) in the present sample.

### 3.3 The Arabic Single Item Trait Empathy Scale (SITES)

The SITES consists of the following item: “I am an empathetic person”, and allows to assess trait empathy [40]. The Arabic version of the SITES was used in this study [41]. Respondents are asked to score the extent to which the statement describes them in a five‐point Likert‐type scale ranging from 1 (“Not very true of me”) to 5 (“Very true of me”).

## 4 Analytic Strategy

No missing responses were recorded for the 9‐SRA items. Some demographic variables (e.g. age and sex) contained missing values, and these were left as is without imputation. To evaluate the factorial structure of the 9‐SRA, we performed a confirmatory factor analysis (CFA) using RStudio, with the "lavaan" and "SemTools" packages [42, 43]. Therefore, parameter estimates were derived using the robust maximum likelihood method estimator, which yields reliable fit statistics in the presence of non‐normal data. The latent factor was identified by constraining the loading of the first item to 1. Model adequacy was judged by several fit indices: root mean square error of approximation (RMSEA; ≤ 0.08), standardized root mean square residual (SRMR; ≤ 0.05), Tucker‐Lewis Index (TLI; ≥ 0.90), and Comparative Fit Index (CFI; ≥ 0.90) (31). Convergent validity was evaluated through the average variance extracted (AVE), with values ≥ 0.50 considered satisfactory. Tests of multivariate normality showed significant departures (Mardia's skewness = 1616.60; *p* < 0.001; Mardia's kurtosis = 79.16; *p* < 0.001).

Measurement invariance by sex was examined through multi‐group CFA. We assessed invariance at the configural, metric, scalar and strict levels. Evidence of invariance was defined as ΔCFI ≤ 0.010 and ΔRMSEA ≤ 0.015 or ΔSRMR ≤ 0.010. when invariance was confirmed, we compared total scores between males and females using the Mann‐Whitney test.

Internal consistency was examined through McDonald's ω and Cronbach's α, considering coefficients above 0.70 to indicate adequate reliability. Spearman's rank correlations (rho) were calculated to explore the associations between altruism and other constructs. Correlation strength was interpreted as small (≈ 0.2), moderate (≈ 0.5) or large (≈ 0.8) [44].

All analyses described above were pre‐specified in the statistical analysis plan, and no additional exploratory subgroup analyses were undertaken. All hypothesis tests were two‐sided with a predefined significance threshold of *p* < 0.05. Exact *p* values are reported according to recommended conventions.

## 5 Results

A total of 615 HCWs participated in the study. The mean age was 28.54 years (SD = 9.08), with 67.6% female participants and 60.7% nurses. Detailed sociodemographic characteristics are summarized in Table 1.

**Table 1:** Sociodemographic information of the participants (*n* = 615).

Variable	Mean ± SD
Age (years)	28.54 ± 9.08
**Variable**	***N* (%)**
Sex	
Males	199 (32.4%)
Females	416 (67.6%)
Marital status	
Single/Divorced/Widowed	169 (27.5%)
Married	446 (72.5%)
Occupation	
Physician	140 (22.8%)
Nurse	373 (60.7%)
Other healthcare worker	102 (16.6%)
Workplace	
Hospital	449 (73.0%)
Primary health care center	166 (27.0%)

Item‐level frequencies for the 9‐SRA are presented in Table 2. On the total score range (9–45), participants obtained a mean 9‐SRA score of 20.00 (SD = 8.00). Using the cut‐off score of ≥ 28, 104 participants (16.0%) were classified as having high altruism.

**Table 2:** Frequency of each item of the 9‐SRA.

Item	Frequency	
	“Never”	“Often” to “Very often”
1. I have given money to a charity	286 (46.0%)	77 (12.5%)
2. I have donated goods or clothes to a charity	203 (33.0%)	85 (13.8%)
3. I have done volunteer work for a charity	221 (35.0%)	92 (14.9%)
4. I have helped carry a stranger's belongings	244 (39.0%)	109 (17.7%)
5. I have made change for someone I did not know	203 (33.0%)	117 (19.0%)
6. I have helped an acquaintance to move houses	255 (41.0%)	89 (14.5%)
7. I have let a neighbor I did not know well borrow an item of some value to me	200 (32.0%)	118 (19.2%)
8. I have offered to help a disabled or elderly stranger across a street	192 (31.0%)	117 (19.0%)
9. I have offered my seat to a stranger who was standing	233 (37.0%)	109 (17.7%)

### 5.1 Confirmatory Factor Analysis of the 9‐SRA Scale

**Figure 1:**
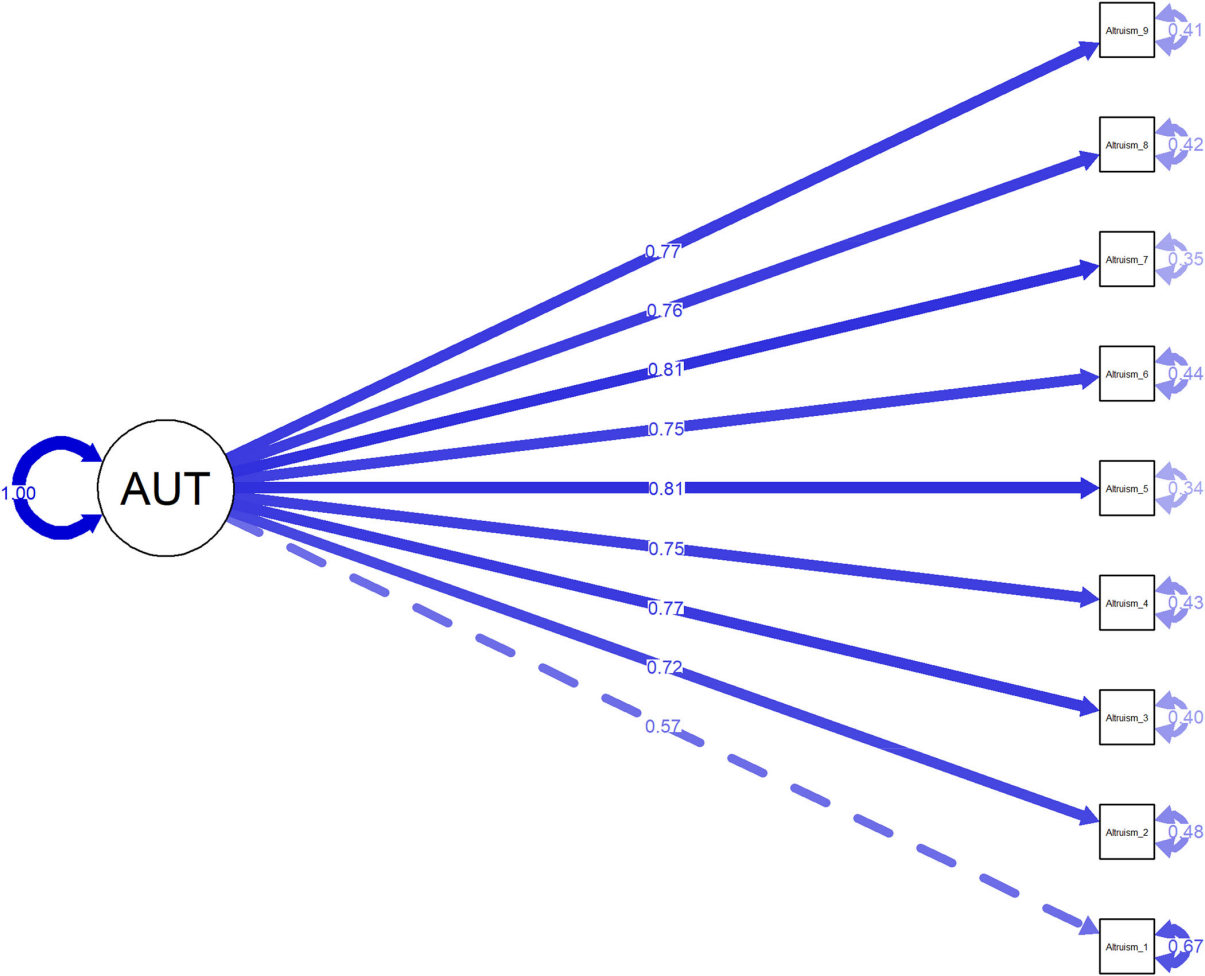
Standardized loading factors of the one‐factor model of the 9‐SRA items in Arabic.

CFA supported the proposed two‐factor structure of the 9‐SRA scale with excellent model fit indices: Robust RMSEA = 0.043 (90% CI [0.035, 0.051]), SRMR = 0.043, Robust CFI = 0.994, Robust TLI = 0.992. All standardized factor loadings were > 0.4 (Figure 1). The scale demonstrated excellent internal consistency (*ω* = 0.92/*α* = 0.92) and satisfactory convergent validity (AVE = 0.56).

### 5.2 Sex Invariance

Multi‐group CFA supported configural, metric and scalar invariance across sex and profession (Table 3). Females had higher altruism scores than males (M = 20.78, SD = 8.23 vs. M = 18.98, SD = 8.63; *t*(613) = −2.50; *p* = 0.013, Cohen's *d* = 0.215).

**Table 3:** Measurement invariance of the altruism scale.

Model	CFI	RMSEA	SRMR	Model comparison	ΔCFI	ΔRMSEA	ΔSRMR
**Model 1: by sex**							
Males	0.927	0.135	0.043				
Females	0.946	0.101	0.044				
Configural	1.000	0.001	0.042				
Metric	1.000	0.001	0.048	Configural vs. metric	< 0.001	< 0.001	0.006
Scalar	0.922	0.110	0.052	Metric vs. scalar	0.078	0.109	0.004
Strict	1.000	0.001	0.054	Scalar vs. strict	0.078	0.109	0.002
**Model 2: by profession**							
Physicians	0.875	0.134	0.062				
Nurses	0.936	0.121	0.043				
Other healthcare professionals	0.957	0.101	0.042				
Configural	0.930	0.121	0.043				
Metric	0.932	0.109	0.050	Configural vs. metric	0.002	0.012	0.007
Scalar	0.928	0.104	0.054	Metric vs. scalar	0.004	0.005	0.004
Strict	0.926	0.097	0.055	Scalar vs. strict	0.002	0.007	0.001

Abbreviations: CFI, comparative fit index; RMSEA, root mean square error of approximation; SRMR, standardized root mean square residual.

### 5.3 Convergent and Concurrent Validity

Altruism scores were positively correlated with empathy (rho = 0.11; *p* = 0.008). Higher altruism was also significantly and moderately associated with higher depression (rho = 0.33; *p* < 0.001) and higher anxiety (rho = 0.36; *p* < 0.001).

### 5.4 Discriminant Validity

The square root of the AVE (= 0.71) exceeded the correlations of the 9‐SRA and both depression and anxiety measures, supporting discriminant validity.

## 6 Discussion

There is a widely accepted assumption that humans are self‐serving in nature, and inherently more inclined to engage in altruistic behavior in challenging situations or when resources are scarce [45]. In this context, HCWs' willingness to care for others' lives when faced with a war that threatens their survival is intriguing, and identifying its potential explaining factors could help inform health systems' response to disasters and develop effective strategies for the recruitment and retention of the health workforce in such extreme conditions. To help advance this field, our study proposed to test the psychometric properties of a brief altruism scale in a sample of HCWs, and to investigate correlations between altruism, depression, anxiety, and empathy in a high‐stress, real‐world setting. Our findings provided preliminary support for the good psychometric qualities of the 9‐SRA scale among HCWs working in a war zone and an Arabic‐speaking context. In addition, altruism correlated positively as expected with depression‐anxiety and empathy scores. These correlations provide ecological insight and are a valuable addition to the literature.

The originally proposed unidimensional model of the 9‐SRA was tested by CFA. The goodness‐of‐fit indices showed that our data adequately fitted this original hypothetical one‐factor model, with excellent internal consistency reliability coefficients (MC Donald's *ω* = 0.92 and Cronbach's α = 0.92). These results were similar to those found in the seminal validation paper by Manzur and Olavarrieta [35], which also demonstrated adequate validity and reliability, with a total Cronbach's alpha coefficient of 0.77 in a sample of American citizens. Moreover, and in line with our hypothesis, the assumption of measurement invariance across sex groups of the Arabic version of the 9‐SRA was supported at the configural, metric, scalar and strict levels. Providing evidence for measurement invariance across sex signifies that the instrument is comparable across respondents of both sexes, and that males and females interpret the question items in a similar way between sex groups [46]. This permits to make accurate and meaningful empirical quantitative comparisons of altruistic disparities between males and females. In this regard, the 9‐SRA differentiated between male and female HCWs in terms of total scores. In particular, it was observed that females reported greater levels of altruistic behaviors compared to males, which was consistent with results from several previous studies. For instance, experimental studies (such as dictator game experiments) reported that females tended to be more altruistic than males [47, 48].

The war in Gaza provides a real‐life ecological context to explore altruists' psychological responses and its correlates in specific disaster circumstances. We found that 9‐SRA scores positively and significantly correlated with scores of depression and anxiety. These results lend support to the recent research findings that contend that, while altruism has long been regarded as protective for psychological health [16, 17, 18], it has been shown to negatively affect mental health under crises situations [19]. This paradox can be explained by the fact that, under extreme conditions, when altruists cannot perform the intended altruistic act to help others or perceive their acts as without benefits in the midst of chaos, they may feel reduced self‐efficacy, helplessness and other negative emotional experiences. Additionally, altruism is generally prompted by feelings of compassion and empathy [49]. On the battlefield, HCWs often work with extremely limited intervention resources and effectiveness, along with extremely low survival rates. In such conditions, altruist HCWs are led to see their patients frightened and dying in pain, which might increase their negative emotions. In sum, and in line with some previous findings, the benefits of altruism seem to be conditioned by the altruists' ability to be caring and effectively helpful to others. For instance, a study performed among Chinese individuals who were self‐isolated during the COVID‐19 found that altruism was significantly linked to increased depressive and anxiety symptoms, and that altruism served as a moderator in worse mental health by heightening negative affect [19]. It was suggested that altruists may experience more anxiety and depression due to their empathy and feelings of helplessness towards people affected by the virus [19]. Likewise, a study by Vieira et al. [50] that was conducted among US residents during COVID‐19 showed that individuals who self‐reported greater everyday altruism experienced more severe acute anxiety. Overall, our results may help advance altruism theories and offer valuable information for researchers, practitioners and policy‐makers regarding the influence of altruism on HCWs' mental health during war.

### 6.1 Practical Implications

The present validation study represents the first preliminary evidence for the potential applicability of the short 9‐item version of the SRA scale in the healthcare psychology field to understand altruist behaviors by HCWs. This might help inform healthcare organizations' efforts to provide healthcare in case of war or disaster when HCWs' personal safety, health and life are threatened, by identifying, understanding and fostering altruistic tendencies among healthcare professionals. The 9‐SRA has the advantage of being a brief self‐report tool assessing altruistic behavior, and being, to date, the only well‐established and validated measure of altruism in the Arabic language. Its brevity allows to diminish the demand placed on survey participants and reduce both response and nonresponse biases, especially when the research is carried out in a disaster setting. Furthermore, findings of the present study contribute in illuminating HCWs' lived experiences of altruism and its correlation with mental health during one of the deadliest wars of all time. However, it is noteworthy that the present validation efforts are context‐specific for HCWs in Gaza, and findings might not be generalizable to broader community populations.

As for future research endeavors in this area, our findings and the new scale made available may facilitate and guide further investigations on the psychological mechanisms of altruism and the role of everyday altruism on mental health responses, especially amid extreme life‐threatening circumstances. Of note, the cross‐sectional nature of our data precludes drawing firm empirical conclusions about the cause‐and‐effect relationship between the study variables. Given the idea that difficult and challenging circumstances can potentially promote, rather than hinder, altruistic behavior [51, 52], future longitudinal and experimental studies are warranted to explore the change of altruistic tendencies in the target population over time during war and post‐war periods. Moreover, further prospective cohort studies are critical for identifying and understanding how altruistic dispositions may influence HCWs' mental health status during a real‐life crisis.

### 6.2 Study Limitations

Despite its originality and contribution to existing knowledge, this study has limitations that need to be discussed. Because of the cross‐sectional design, our findings were inconclusive to determine a potential causal link between levels of altruistic behavior and changing mental health over time. Additionally, sampling issues need to be carefully borne in mind, as the snowball sampling approach used to recruit participants, although highly practical in a war setting, can lead limited generalizability and representativeness. Moreover, the majority of participant HCWs were females (67.6%), which may have skewed our findings to some extent. Another limitation to this study is the lack of a control or comparison group. The validation of the 9‐SRA scale in samples with varying levels of altruism (e.g., HCWs at war vs. at peace) could allow for more heterogeneity in the data, for comparisons to be made across different sample types, and for further insights into the applicability of the scale in different settings and contexts. Furthermore, an important consideration to be taken into account by users of the 9‐SRA is that it might be subject to social desirability bias due to its self‐report nature. Besides, one salient point to consider is that the 9‐SRA used as an indicator of altruism does not specify a time period for when the altruistic acts were undertaken, and might have included behaviors that did not necessarily occur during the ongoing war. However, all nine items of the scale describe behaviors that can be performed during war‐time. A potential limitation is that, given the extraordinary demands on healthcare workers during the war, altruism levels were likely to be high, leading to a restricted range of responses, which might inflate internal consistency numbers while reducing discriminant validity. Future studies should examine this scale in more heterogenous samples in different contexts.

## 7 Conclusion

Our research offers robust evidence in terms of reliability and validity to support the use of the 9‐SRA instrument to evaluate self‐reported altruistic behavior among Arabic‐speaking HCWs, especially in the context of war. Moreover, our study adds to the literature by reporting positive links of altruism to depression‐anxiety and empathy. To enhance knowledge of how altruism manifests in HCWs and to what extent it could impact their mental health, clinicians and researchers should consider using the self‐report 9‐SRA in routine clinical and research practice.

## Author Contributions

Conceptualization: Feten Fekih‐Romdhane. Methodology: Feten Fekih‐Romdhane. Software: Souheil Hallit. Data curation: Kamel Jebreen, Inad Nawajah, Tasnim Swaitti, Eqbal Radwan, Bushra M. Hammad, Mohammed Jebreen, Etimad Alattar, Walaa Safi. Investigation: Feten Fekih‐Romdhane. Validation: Souheil Hallit. Formal analysis: Souheil Hallit. Supervision: Feten Fekih‐Romdhane. Project administration: Feten Fekih‐Romdhane. Resources: Feten Fekih‐Romdhane. Writing – original draft: Feten Fekih‐Romdhane and Souheil Hallit. Writing – review and editing: Feten Fekih‐Romdhane, Kamel Jebreen, Inad Nawajah, Tasnim Swaitti, Eqbal Radwan, Wafa Kammoun‐Rebai, Bushra M. Hammad, Mohammed Jebreen, Etimad Alattar, Walaa Safi, Sahar Obeid, and Souheil Hallit.

## Conflicts of Interest

The authors declare no conflicts of interest.

## Transparency Statement

The lead author Feten Fekih‐Romdhane, Souheil Hallit affirms that this manuscript is an honest, accurate, and transparent account of the study being reported; that no important aspects of the study have been omitted; and that any discrepancies from the study as planned (and, if relevant, registered) have been explained.

## Data Availability

The data that support the findings of this study are available from the corresponding author but restrictions apply to the availability of these data, which were used under license for the current study, and so are not publicly available. Data are however available from the authors upon reasonable request and with permission of the ethics committee.
